# MacroH2A1 Immunoexpression in Breast Cancer

**DOI:** 10.3389/fonc.2020.01519

**Published:** 2020-08-21

**Authors:** Giuseppe Broggi, Veronica Filetti, Antonio Ieni, Venerando Rapisarda, Caterina Ledda, Ermanno Vitale, Silvia Varricchio, Daniela Russo, Claudia Lombardo, Giovanni Tuccari, Rosario Caltabiano, Carla Loreto

**Affiliations:** ^1^Section of Anatomic Pathology, Department Gian Filippo Ingrassia, University of Catania, Catania, Italy; ^2^Human Anatomy and Histology, Department of Biomedical and Biotechnology Sciences, University of Catania, Catania, Italy; ^3^Department of Human Pathology in Adult and Developmental Age “Gaetano Barresi”, Section of Pathology, University of Messina, Messina, Italy; ^4^Occupational Medicine, Department of Clinical and Experimental Medicine, University of Catania, Catania, Italy; ^5^Department of Advanced Biomedical Sciences, Pathology Unit, University of Naples Federico II, Naples, Italy

**Keywords:** breast cancer, macroH2A1, immunohistochemistry, metastases, prognosis

## Abstract

MacroH2A1 has two splice isoforms, macroH2A1.1 and macroH2A1.2, that have been studied in several form of cancer. In the literature there are not many scientific papers dealing with the role of macroH2A1 in breast cancer. Breast cancer is the most frequent form of malignancy in females. It tend to metastasize to the bone in ~70% of patients. Despite treatment, new bone metastases will still occur in 30–50% of cases with advanced disease. Overall 5-year survival after the diagnosis of bone metastasis is ~20%. Osteoclasts and osteoblasts of the bone microenvironment are engaged by soluble factors released by neoplastic cells, resulting in bone matrix breakdown. This malfunction enhances the proliferation of the cancer cells, creating a vicious cycle. We investigated immunohistochemical expression of macroH2A1 in primitive breast cancer, focusing on the comparison of metastatic and non-metastatic cases. Furthermore, the immunohistochemical expression of macroH2A1 has been evaluated both in all cases of nodal metastases and in distant metastases. Our data demonstrated that macroH2A1 expression was higher expressed in metastatic breast cancer (77%) vs. non-metastatic breast cancer (32%). Also in analyzed metastases cases, a high macroH2A1 expression was detected: 85 and 80% in nodal and distant metastases cases, respectively. These results supported the fact that macroH2A1 is more highly expressed in breast cancer with worst prognosis.

## Introduction

Breast cancer actually represents a global health challenge and is still one of the most relevant topics in biomedical research. Worldwide, it is the most frequent form of malignancy in females and incidence and mortality rates are predicted to significantly increase in years to come. The incidence of breast cancer with ~1,700,000 new cases each year remains alarmingly high; and these data suggest that several steps taken forward so far in the prevention setting are not yet sufficient (1–3). Both incidence and mortality rates are expected to be disproportionately high in developing countries and are estimated to reach 55% increased incidence and 58% greater mortality in 20 years (3). Breast cancer spreads to the bone in ~70% of cases with advanced tumor. Despite treatment, new bone metastases will still occur in 30–50% of patients. Only 20% of patients with bone metastases survive 5 years after the diagnosis of bone metastasis (4).

Substantial progress has been made to understand histone H2A variants, their correlation to normal cellular function and to cancer development and progression (5). MacroH2A1 is a histone variant consisting of C-terminal macro domain (6), it is enhanced by the inactive X chromosome (X1) in mammals, infact it plays a fundamental role in gene expression inhibition due to X inactivation (7–9). Although the majority of studies of macroH2A1 demonstrate its role in transcriptional repression with consequent inactivation of genes encoding the factors that affect osteoclastogenesis and metastatic spread in breast cancer cells (10) recent studies revealed that macroH2A1 protects a subset of its target genes from silencing (11–20). Thus, macroH2A1 also participates in signal-induced gene activation (11–20) because is also found in large chromatin domains marked by acetylations (12, 14, 15, 19). Indeed, genes present in macroH2A1 domains can be either positively or negatively regulated by macroH2A1 depending of the specific chromatin microenvironments (12).

MacroH2A1 has two splice isoforms, macroH2A1.1 and macroH2A1.2, that have been studied in several form of cancer (7, 10, 12, 21–26), but in breast cancer the function of macroH2A1 has not been much evaluated.

We investigated the immunohistochemical expression of the human histone variant macroH2A1 in 54 primitive breast cancers, both in presence and in absence of nodal metastases. The immunohistochemical expression of macroH2A1 has been evaluated in nodal and distant metastases, too. This work focuses attention about the correlation between primitive tumors and metastasis and about the relationship between macroH2A1 expression and prognosis in terms of metastatic risk, in order to recognize a potential biomarker capable of predicting the natural history and the prognosis of breast cancer.

## Materials and Methods

### Patients and Tissue Samples

Histologic specimens of 54 cases of primitive breast cancer treated with quadrantectomy and sentinel limphnode biopsy followed by axillary limphadenectomy if sentinel node was positive, were retrospectively analyzed. Furthermore, all cases of nodal metastases (*n* = 26) and distant metastases (*n* = 5) from the same patients were included in the analysis. Formalin-fixed and paraffin-embedded tissue specimens were obtained from the files of the Sections of Anatomic Pathology of the Department “Gian Filippo Ingrassia,” University of Catania, and of the Department of Human Pathology in Adult and Developmental Age “Gaetano Barresi,” University of Messina. We adopted the following exclusion criteria in the choice of the cases: (i) it was no possible to obtain additional slides from paraffin blocks for immunohistochemical evaluation; (ii) no representative neoplastic tissue was contained in paraffin blocks.

Because of the retrospective nature of the study, no written informed consent was necessary; the research protocols were conformed to the ethical guidelines of the Declaration of Helsinki.

Fifty-four female patients at an average age of 58 years (range 28–81) were part of the study. In particular, the patients with nodal metastases at the time of surgery were 26 at an average age of 57.5 ± 10.1 years (range 40–81). Conversely, patients with non-metastatic disease were 28 at an average age of 59.1 ±12.6 years (range 28–79) (Table 1). The five distant metastases were located to the bone (*n* = 1) and to the brain (*n* = 4).

**Table 1:** Demographics, tumor parameters, histotypes, presence/absence of metastasis, molecular subtype, and macroH2A1 expression in primary breast cancer (*n* = 54).

**Age (years)**	**Histologic type**	**TNM**	**Nodal metastasis**	**Molecular subtype**	**MacroH2A.1 IS**	**MacroH2A.1 ES**	**MacroH2A.1 IRS**
47	Invasive ductal carcinoma of NST (G2)	pT1c N3a	Yes	Luminal B/HER2 –	0	0	0
63	Invasive ductal carcinoma of NST (G2)	pT4d N2a	Yes	Luminal B/HER2 –	2	2	4
39	Invasive ductal carcinoma of NST (G3)	pT1c N0	No	HER2 +	1	1	1
75	Invasive tubular carcinoma (G1)	pT1c N0	No	Luminal A	2	2	4
53	Invasive lobular carcinoma (G2)	pT2 N0	No	Luminal A	1	2	2
63	Invasive ductal carcinoma of NST (G2)	pT1c N3a	Yes	Luminal B/HER2 –	2	2	4
44	Invasive ductal carcinoma of NST (G2)	pT1c N0	No	Luminal B/HER2 –	2	2	4
63	Invasive ductal carcinoma of NST (G1)	pT1b N0	No	Luminal A	1	1	1
74	Invasive ductal carcinoma of NST (G2)	pT1bN1mi	Micrometastasis	Luminal B/HER2 –	2	4	8
54	Invasive ductal carcinoma of NST (G2)	pT1c N0	No	Luminal A	2	4	8
46	Invasive ductal carcinoma of NST (G3)	pT2 N2a	Yes	HER2 +	3	3	9
50	Invasive tubular carcinoma (G1)	pT3 N0	No	Luminal A	2	2	4
56	Invasive ductal carcinoma of NST (G2)	pT1b N0	No	Luminal B/HER2 –	1	2	2
45	Invasive ductal carcinoma of NST (G2)	pT1c N0	No	Luminal A	1	1	1
57	Invasive ductal carcinoma of NST (G3)	pT2 N1a	Yes	Luminal B/HER2 –	3	3	9
59	Invasive ductal carcinoma of NST (G3)	pT2 N1a	Yes	Luminal B/HER2 –	3	4	12
68	Invasive ductal carcinoma of NST (G2)	pT1b N0	No	Luminal A	2	1	2
64	Invasive ductal carcinoma of NST (G2)	pT1c N0	No	Luminal B/HER2 –	1	3	3
40	Invasive ductal carcinoma of NST (G3)	pT2 N1a	Yes	Luminal B/HER2 –	3	3	9
65	Invasive lobular carcinoma (G2)	pT1c N2a	Yes	Luminal B/HER2 –	3	4	12
67	Invasive ductal carcinoma of NST (G2)	pT1c N0	No	Luminal A	1	2	2
28	Invasive ductal carcinoma of NST (G1)	pT1c N0	No	Luminal A	2	2	4
55	Invasive ductal carcinoma of NST (G3)	pT2 N3b	Yes	Luminal B/HER2 –	0	0	0
63	Invasive ductal carcinoma of NST (G2)	pT1c N0	No	Luminal B/HER2 –	3	4	12
81	Invasive ductal carcinoma of NST (G3)	pT1c N1a	Yes	HER2 +	2	4	8
46	Invasive ductal carcinoma of NST (G3)	pT1c N1a	Yes	Triple negative	2	4	8
56	Invasive lobular carcinoma (G3)	pT2 N3b	Yes	Luminal B/HER2 +	2	4	8
61	Invasive ductal carcinoma of NST (G2)	pT1b N2a	Yes	Luminal B/HER2 –	1	1	1
69	Invasive ductal carcinoma of NST (G2)	pT1c N2a	Yes	Luminal B/HER2 +	3	3	9
54	Invasive ductal carcinoma of NST (G3)	pT1c N0	No	Luminal B/HER2 –	2	3	6
62	Invasive ductal carcinoma of NST (G2)	pT1c N1a	Yes	Luminal B/HER2 –	2	4	8
61	Invasive ductal carcinoma of NST (G3)	pT1c N2a	Yes	Luminal B/HER2 +	3	3	9
65	Invasive ductal carcinoma of NST (G3)	pT2 N2a	Yes	Luminal B/HER2 +	3	4	12
43	Invasive tubular carcinoma (G2)	pT2 N0	No	Luminal B/HER2 +	2	3	6
49	Invasive tubular carcinoma (G1)	pT1b N0	No	Luminal A	2	3	6
78	Invasive ductal carcinoma of NST (G2)	pT1c N1ami	Micrometastasis	Luminal B/HER2 +	1	2	2
67	Invasive ductal carcinoma of NST (G2)	pT2 N0	No	Luminal A	2	4	8
63	Invasive ductal carcinoma of NST (G3)	pT2 N1a	Yes	HER2 +	2	4	8
49	Invasive tubular carcinoma (G2)	pT1c N1a	Yes	Luminal B/HER2 +	3	4	12
67	Invasive tubular carcinoma (G3)	pT1c N0	No	Luminal A	1	2	2
63	Invasive lobular carcinoma (G3)	pT2 N0	No	Luminal A	3	4	12
71	Invasive ductal carcinoma of NST (G2)	pT1c N0	No	Luminal A	2	3	6
53	Invasive ductal carcinoma of NST (G3)	pT1c N2	Yes	Triple negative	2	3	6
79	Invasive tubular carcinoma (G1)	pT1c N0	No	Luminal A	3	4	12
47	Invasive ductal carcinoma of NST (G3)	pT3 N3	Yes	Luminal B/HER2 –	2	4	8
59	Invasive lobular carcinoma (G2)	pT2 N0	No	Luminal A	2	2	4
52	Invasive ductal carcinoma of NST (G3)	pT1c N2a	Yes	Luminal B/HER2 +	3	4	12
50	Invasive lobular carcinoma (G3)	pT2 N1ami	Micrometastasis	Luminal A	3	4	12
60	Invasive ductal carcinoma of NST (G2)	pT1c N0	No	Luminal B/HER2 +	3	4	12
71	Invasive ductal carcinoma of NST (G2)	pT1c N0	No	Luminal A	3	3	9
56	Invasive ductal carcinoma of NST (G3)	pT2 N2b	Yes	Luminal A	3	3	9
76	Invasive ductal carcinoma of NST (G3)	pT2 N1a	Yes	Luminal B/HER2 –	3	4	12
64	Invasive ductal carcinoma of NST (G2)	pT2 N1a	Yes	Luminal A	2	4	8
40	Invasive ductal carcinoma of NST (G3)	pT2 N1a	Yes	Triple negative	3	3	9

Three patients with nodal micrometastasis (> 0.2 mm and < 2 mm in its greatest diameter, according to the 8th Edition of TNM Classification) were considered as non-metastatic. Regarding histotypes, 41 cases were classified as invasive ductal carcinomas of no special type, seven cases as tubular carcinomas and six as invasive lobular carcinomas. According with the Elston-Ellis grading (based on tubule formation, mitotic count and nuclear pleomorphism), ductal carcinomas were graded as low grade (G1, *n* = 4), intermediate grade (G2, *n* = 14) and high grade (G3, *n* = 10). Based on nuclear grading, lobular carcinomas were classified as G2 (*n* = 2) and G3 (*n* = 1). Considering the 8th Edition of TNM classification, 26 cases of breast cancer with nodal metastases were staged as follows: pT1b N2a (*n* = 1, 3.85%), pT1c N1a (*n* = 4, 15.38%), pT1c N2 (*n* = 1, 3.85%), pT1c N2a (*n* = 4, 15.38%), pT1c N3a (*n* = 2, 7.69%), pT2 N1a (*n* = 7, 26.92%), pT2 N2a (*n* = 2, 7.69%), pT2 N2b (*n* = 1, 3.85), pT2 N3b (*n* = 2, 7.69%), pT3 N3 (*n* = 1, 3.85), and pT4d N2a (*n* = 1, 3.85%). TNM staging in 28 non-metastatic mammary neoplasms was: pT1b N0 (*n* = 4, 14.29%), pT1b N1mi (*n* = 1, 3.57%), pT1c N0 (*n* = 15, 53.57%), pT1c N1ami (*n* = 1, 3.57%), pT2 N0 (*N* = 5, 17.86%), pT2 N1ami (*n* = 1, 3.57%), and pT3 N0 (*N* = 1, 3.57%). Finally, based on original pathologic report, metastatic cases were classified in molecular subtypes as follows: luminal A (*n* = 2, 7.69%), luminal B/HER-2 + (*n* = 7, 26.92%), luminal B/HER-2–(*n* = 11, 42.31%), HER-2 + (*n* = 3, 11.54%), triple negative/basal like (*n* = 3, 11.54%); as regards non-metastatic patients: luminal A (*n* = 18, 64.29%), luminal B (*n* = 2, 7.14%), luminal B/HER-2 + (*n* = 3, 10.71%), luminal B/HER-2–(*n* = 4, 14.29%), HER-2 + (*n* = 1, 3.57%).

### Immunohistochemistry

Histologic specimens were treated as previously reported (27–29). Briefly, the deparaffinized slides underwent pretreatment with 10 mg/mL of ovalbumin in phosphate-buffered saline (PBS) followed by 0.2% biotin- PBS, each for 15 min at room temperature, and they were rinsed for 20 min with PBS (Bio-Optica, Milan, Italy) in order to obtain the reduction of the non-specific staining caused by endogenous biotin. The antigenic unmasking was obtained by microwave pretreatment. Then the slides were incubated overnight at 4 °C with rabbit polyclonal anti-macroH2A1 antibody (ab37264; Abcam, Cambridge, UK) diluted 1:200 in PBS (Sigma, Milan, Italy). Sections were counterstained with hematoxylin, dehydrated, mounted (Zymed Laboratories, San Francisco, CA, USA), and observed with a light microscope (Carl Zeiss, Oberkochen, Germany).

The evaluation of the immunohistochemical studies was performed separately by six pathologists (AI, GT, RC, DR, SS and GB) without access to clinical and other pathologic data of the patients.

MacroH2A1 was considered as positive if brown chromogen was observed in the cellular nucleus. Non pathologic skin was used as a positive control, while we obtained a negative control omitting the primary antibody.

The evaluation of both the intensity of the staining and the extent of positive cells was performed by light microscopy as previously reported (21, 30, 31). The intensity of staining (IS) was subclassified into four levels (0–3): absence of staining = 0, mild staining = 1, moderate staining = 2, and strong staining = 3. Similarly, five levels of extent score (ES), the percentage of immunoreactive cells, were identified: <5% (0), 5%−30% (+), 31%−50% (++), 51%−75% (+++), and >75% (++++). ES was evaluated at 200× magnification. The intensity reactivity score (IRS) was obtained multiplying the intensity of staining (IS) and the extent score (ES): if the IRS was ≤ 6, the macroH2A1 expression was assumed as “low” (L-IRS), while “high” (H-IRS) in presence of an IRS >6.

### Statistical Analysis

The data were plotted using Prism for Windows v 6.07 (Graphpad Software; CA, USA).

## Results

**Figure 1:**
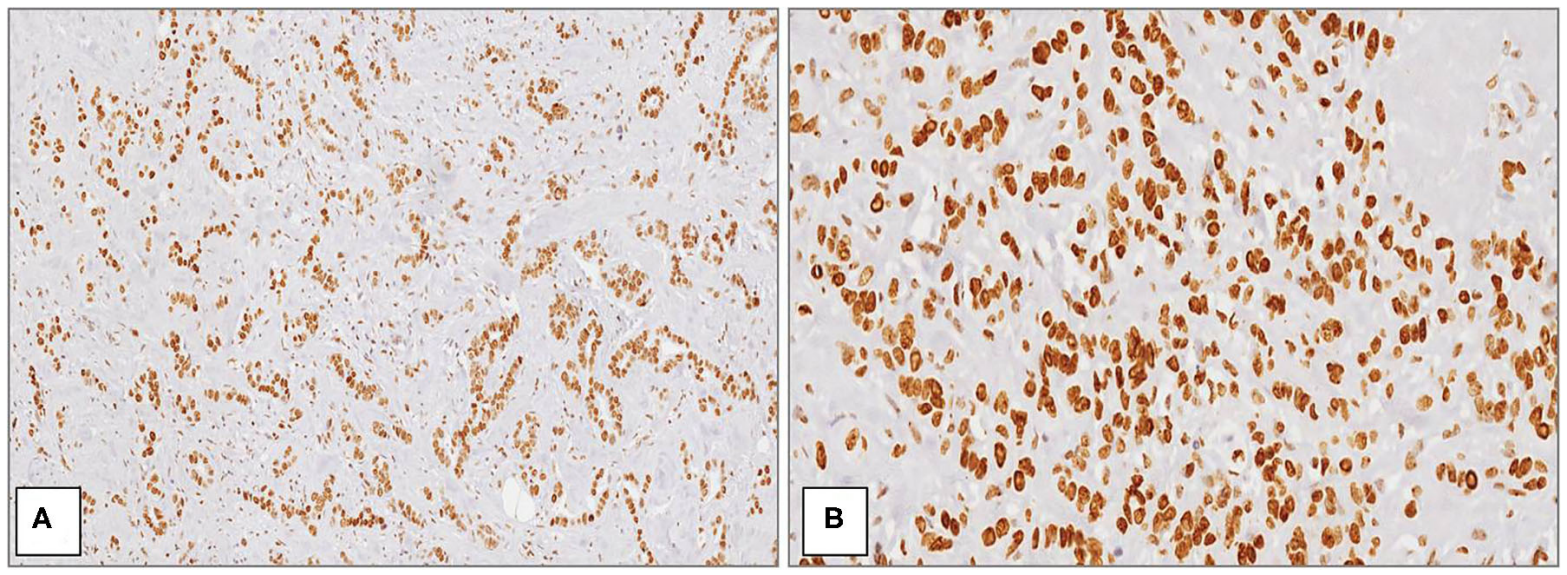
MacroH2A1 in breast cancer. Strong and diffuse nuclear positivity in high grade (G3) ductal carcinoma at medium **(A)** and high magnification **(B)** [Immunoperoxidase stain; original magnification 100× **(A)** and 200× **(B)**].

**Figure 2:**
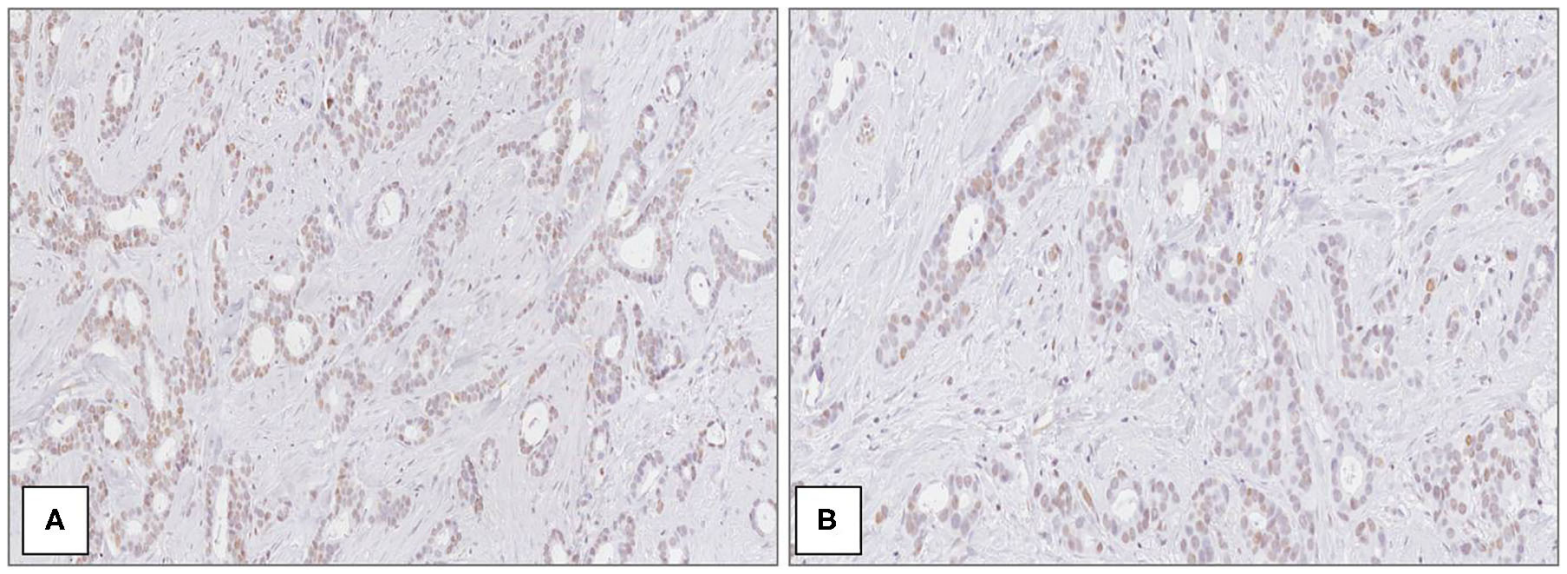
MacroH2A1 in breast cancer. Mild and heterogeneous nuclear positivity in low grade (G1) ductal carcinoma at medium **(A)** and high magnification **(B)** [Immunoperoxidase stain; original magnification 100× **(A)** and 200× **(B)**].

The average age of patients was 58.3 ± 11.4 years in all cases. Considering the whole group (*n* = 54), macroH2A1 H-IRS was observed in 29 (54%) primitive breast cancers (Figure 1) and L-IRS in 25 (46%) (Figure 2).

**Figure 3:**
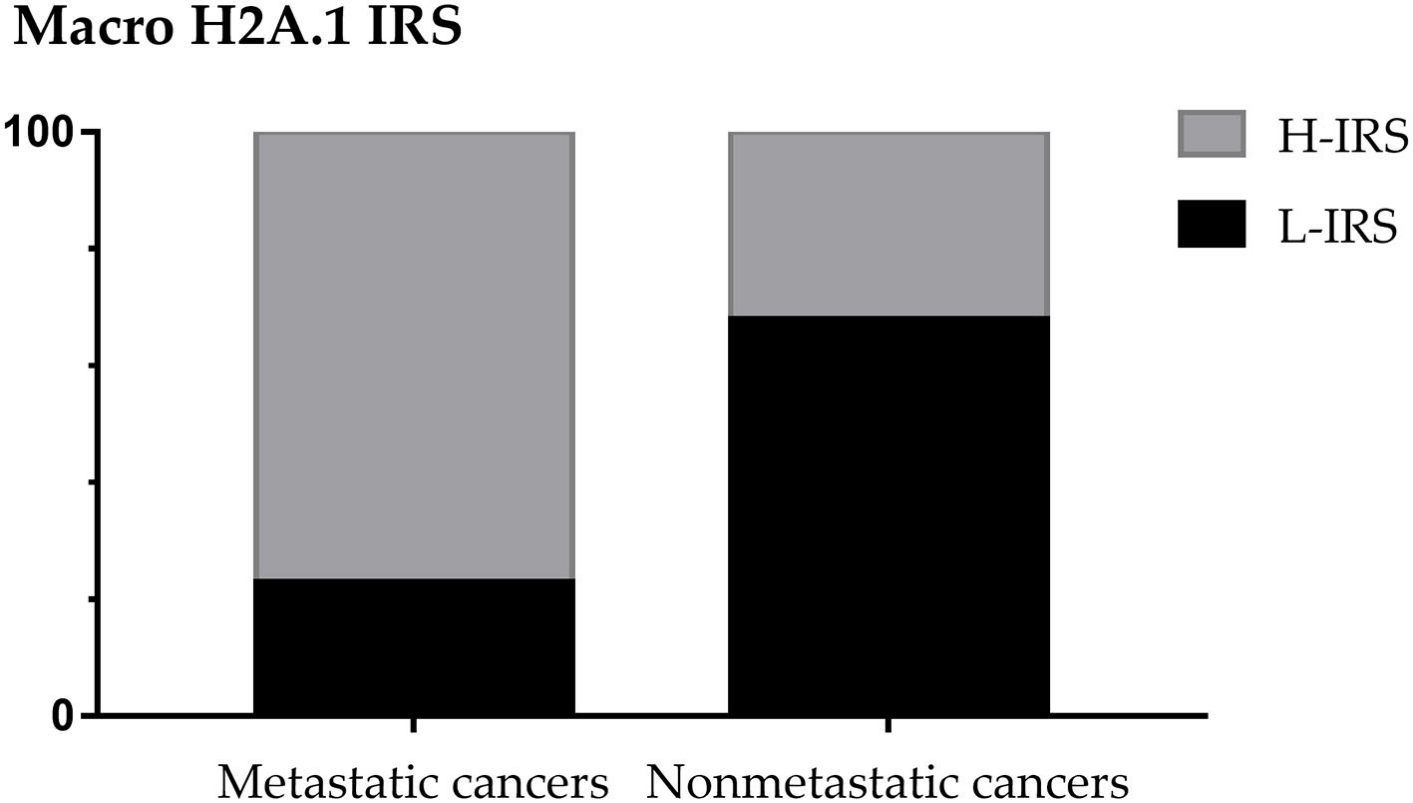
MacroH2A1 Intensity Reactivity Score (IRS) in metastatic and non-metastatic cancers.

The 48% of patients showed the presence of metastases, considering the patients with micrometastases as non-metastatic. In 28 non-metastatic primary breast cancers, H-IRS was observed in nine cases (32%) and L-IRS in 19 (68%) while in 26 metastatic primary breast cancers, H-IRS was observed in 20 (77%) breast cancers and L-IRS in 6 (23%) (Figure 3).

**Figure 4:**
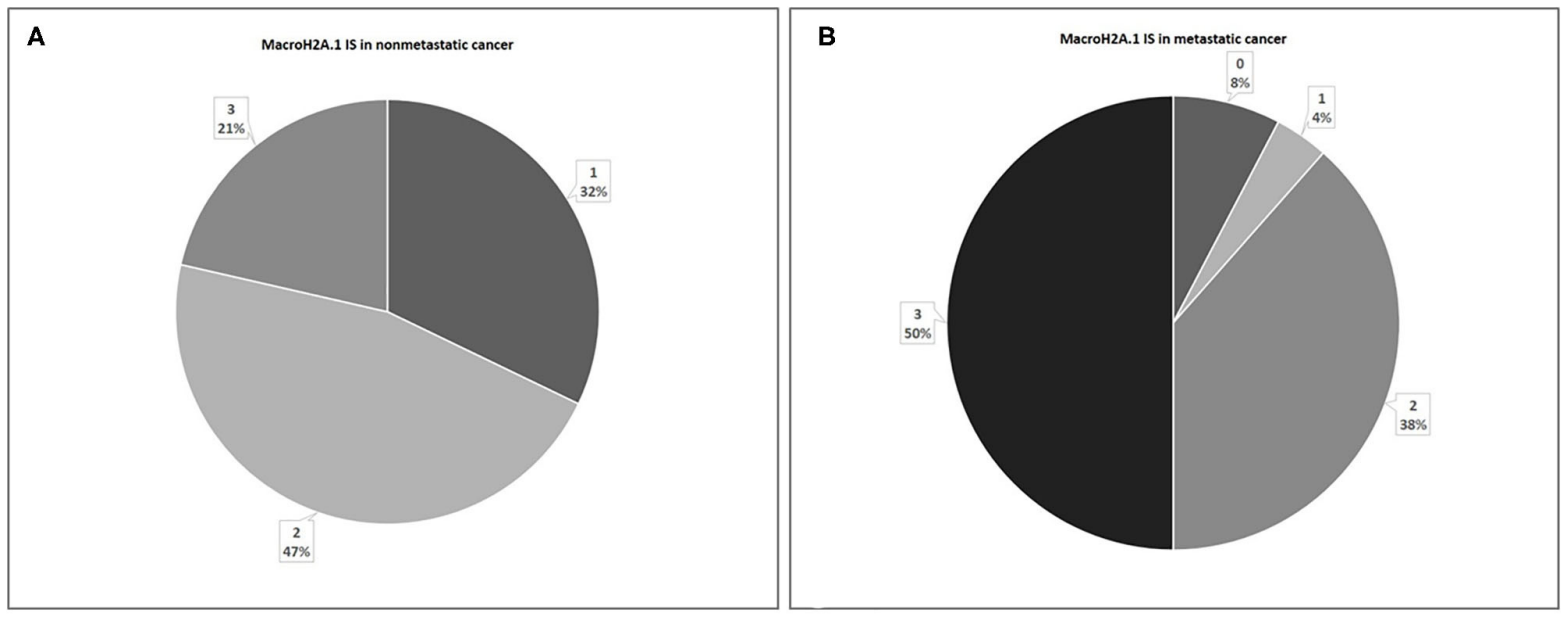
Levels of MacroH2A.1 Intensity of Staining (IS) in non-metastatic **(A)** and metastatic **(B)** breast cancer.

In 28 non-metastatic primary breast cancers, macroH2A1 IS was intermediate in 13 cases (47%) and mild in nine cases (32%), while in six cases (21%) intense immunoreactivity was observed (Figure 4A). In 26 metastatic primary breast cancers, IS was intermediate in 10 cases (38%), mild in only one case (4%), while in 50% of cases intense immunoreactivity was observed. In two cases (8%) no immunoreactivity was observed (Figure 4B).

**Figure 5:**
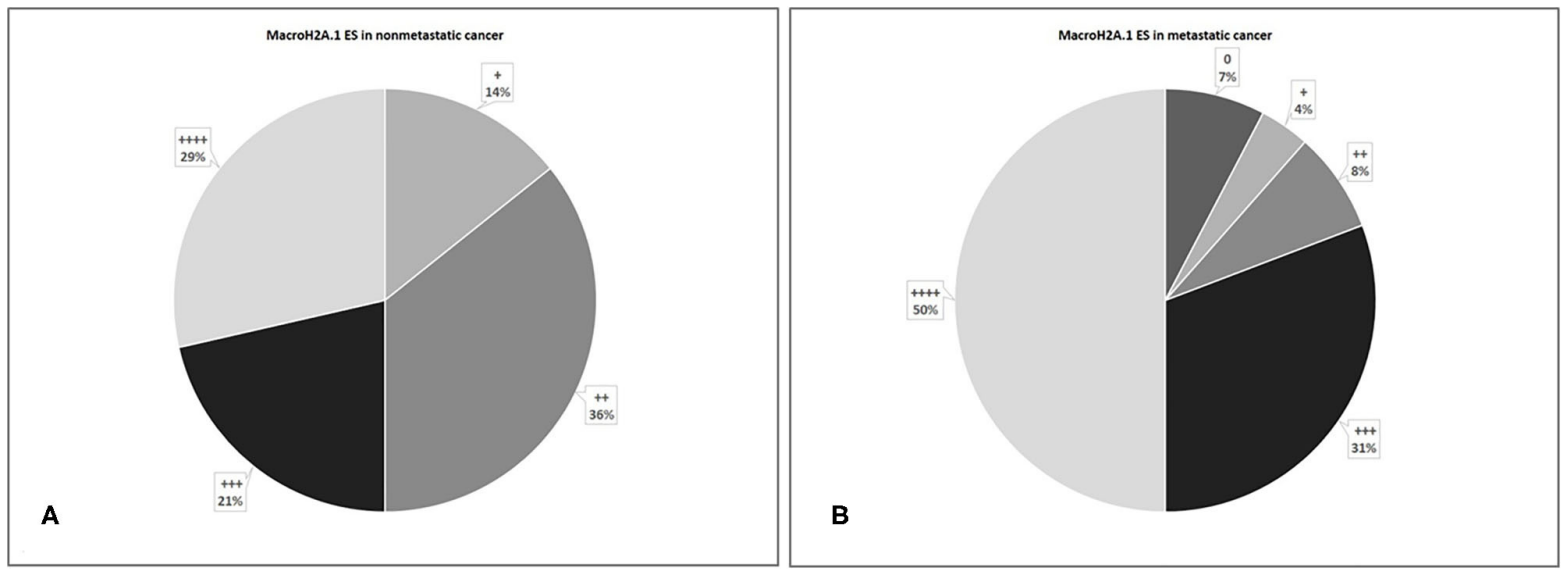
Levels of MacroH2A.1 Extent Score (ES) in non-metastatic **(A)** and metastatic **(B)** breast breast cancer.

In 28 non-metastatic primary breast cancers, macroH2A1 ES was >75% in 8 cases (29%), 51–75% in 6 cases (21%), 31–50% in 10 cases (36%), and 5–30% in 4 cases (14%) (Figure 5A). In 26 metastatic primary breast cancers, ES was >75% in 13 cases (50%), 51–75% in eight cases (31%), 31–50% in two cases (8%), 5–30% in only one case (4%). Furthermore, two cases of macroH2A1 ES <5% (7%) was verified (Figure 5B).

A correlation analysis between the expression of MacroH2A.1 and the clinical-pathological data other than the presence of metastases was carried out. Regardless of the presence/absence of metastasis, macroH2A.1 H-IRS was observed in 31% of invasive ductal carcinoma (G2), in 48% of invasive ductal carcinoma (G3), in 3% of invasive tubular carcinoma (G1), in 3% of invasive tubular carcinoma (G2), in 3% of invasive lobular carcinoma (G2), and in 10% of invasive lobular carcinoma (G3). Regarding macroH2A.1 L-IRS was observed in 8% of invasive ductal carcinoma (G1), in 48% of invasive ductal carcinoma (G2), in 16% of invasive ductal carcinoma (G3), in 12% of invasive tubular carcinoma (G1), in 4% of invasive tubular carcinoma (G2), in 4% of invasive tubular carcinoma (G3), and in 8% of invasive lobular carcinoma (G2).

Focusing on the size of the primary tumor, macroH2A.1 H-IRS was observed in 3% of tumors between 0.6 and 1.0 cm in size (T1b), in 45% of tumors between 1.1 and 2.0 cm in size (T1c), in 48% of tumors >2.0 cm but not more than 5.0 cm in the maximum size (T2), and in 3% of tumors >5.0 cm in the maximum size (T3). MacroH2A.1 L-IRS was observed in 20% of tumors between 0.6 and 1.0 cm in size (T1b), in 56% of tumors between 1.1 and 2.0 cm in size (T1c), in 16% of tumors >2.0 cm but not more than 5.0 cm in the maximum size (T2), in 4% of tumors >5.0 cm in the maximum size (T3), and in 4% of inflammatory carcinoma (T4d).

Focusing on the molecular subtype of cancer, macroH2A.1 H-IRS was observed in 28% of luminal A, in 31% of luminal B/HER2–, in 24% of luminal B/HER2 +, in 10% of HER2 +, and in 7% of triple negative/basal like. MacroH2A.1 L-IRS was observed in 48% of luminal A, in 8% of luminal B, in 28% of luminal B/HER2–, in 8% of luminal B/HER2 +, in 4% of HER2 +, and in 4% of triple negative/basal like.

**Figure 6:**
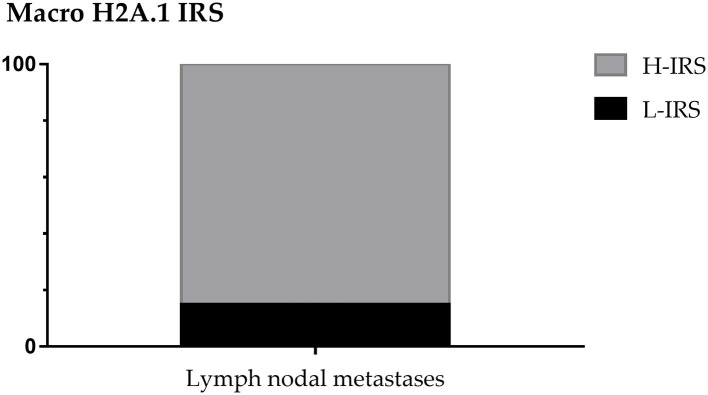
MacroH2A1 Intensity Reactivity Score (IRS) in nodal metastases.

About nodal metastases macroH2A.1 H-IRS was observed in 22 cases (85%) and L-IRS in only 4 cases (15%) (Figure 6).

**Figure 7:**
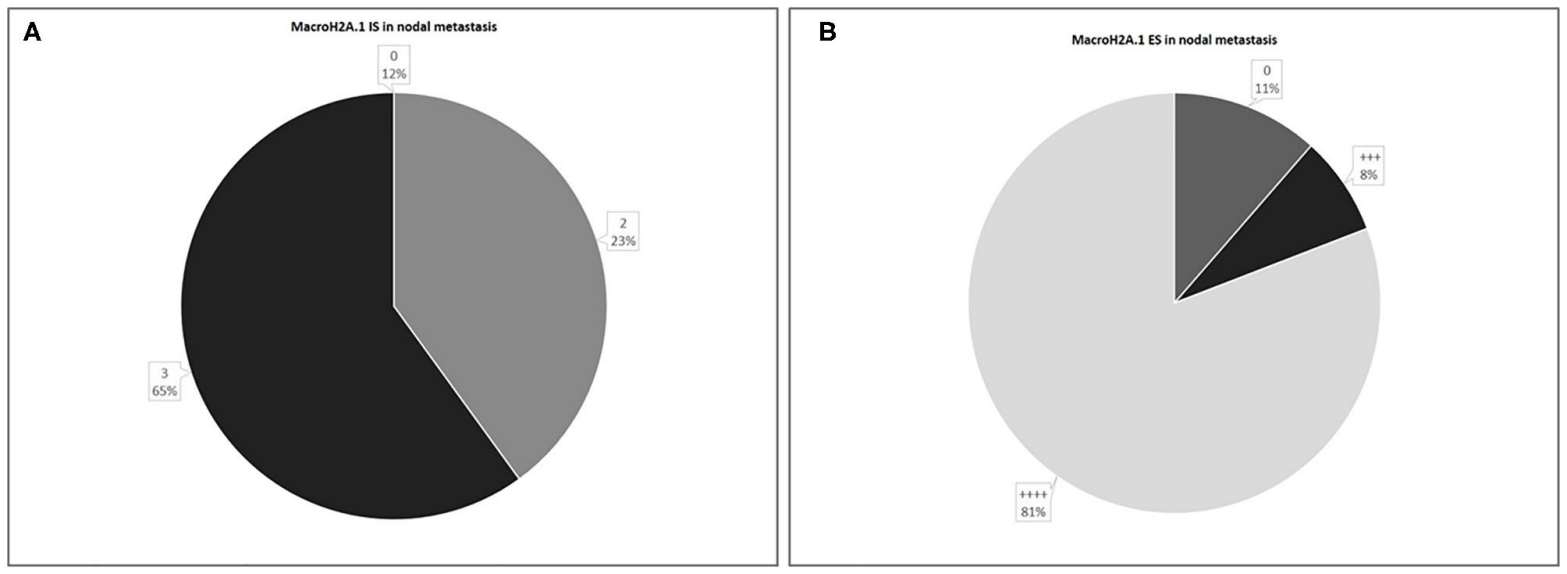
Levels of MacroH2A.1 Intensity of Staining (IS) **(A)** and Extent Score (ES) **(B)** in nodal metastases.

In 26 nodal metastases, macroH2A1 IS was intermediate in 6 cases (23%), intense in 17 cases (65%), while in only 3 cases (12%) no immunoreactivity was observed (Figure 7A). MacroH2A1 ES was >75% in 21 cases (81%), 51–75% in 2 cases (8%), and <5% in 3 cases (11%) (Figure 7B).

**Figure 8:**
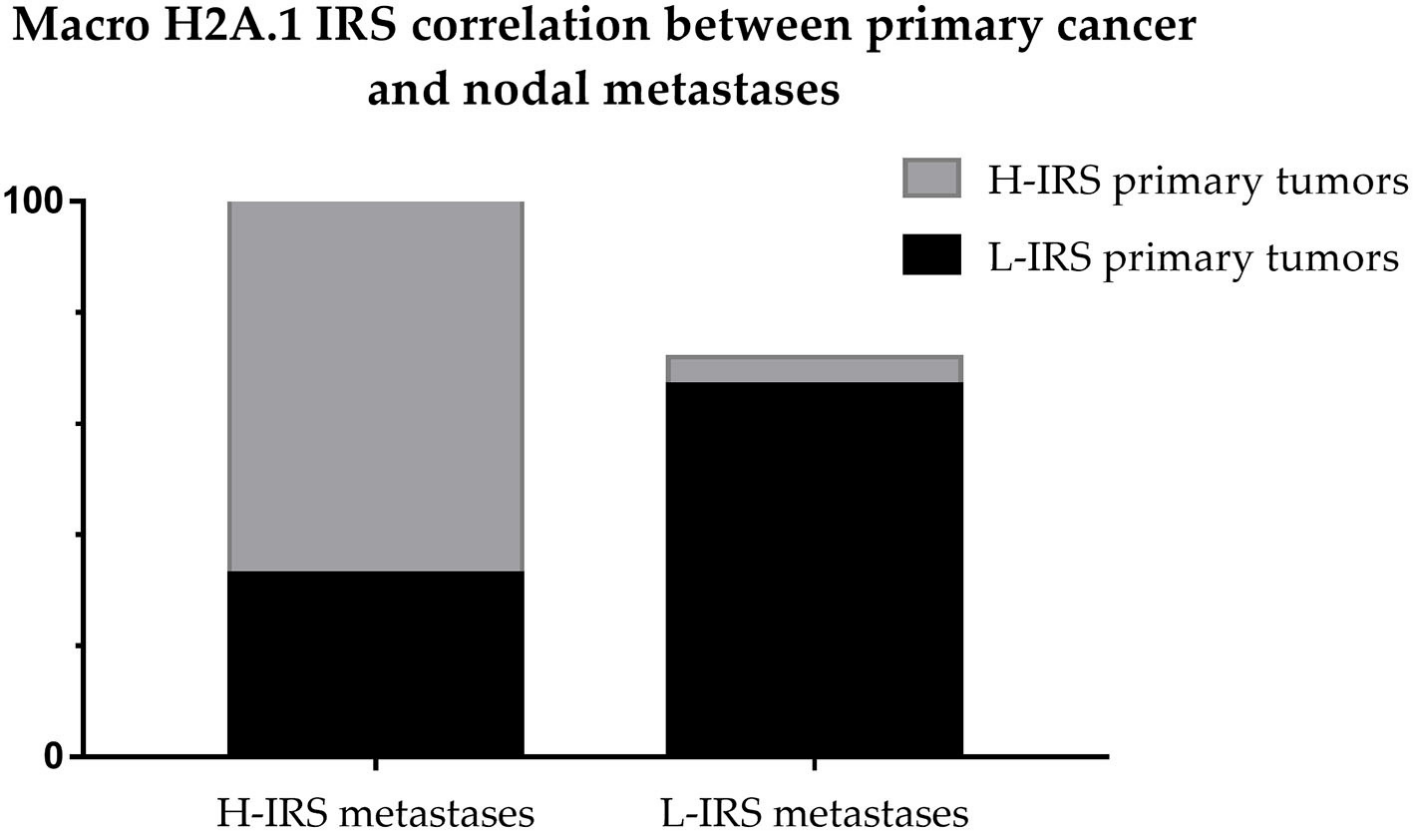
Correlation between Intensity Reactivity Score (IRS) of primitive breast cancers and nodal metastases.

The cases that showed H-IRS in primary breast cancers presented macroH2A1 H-IRS in 21 correlated metastasis cases (95%) and macroH2A1 L-IRS in only one metastasis case (5%). The cases that showed L-IRS in primary breast cancers presented macroH2A1 H-IRS in 2 related metastasis cases (33%) and macroH2A1 L-IRS in 4 metastasis cases (67%) (Figure 8).

**Figure 9:**
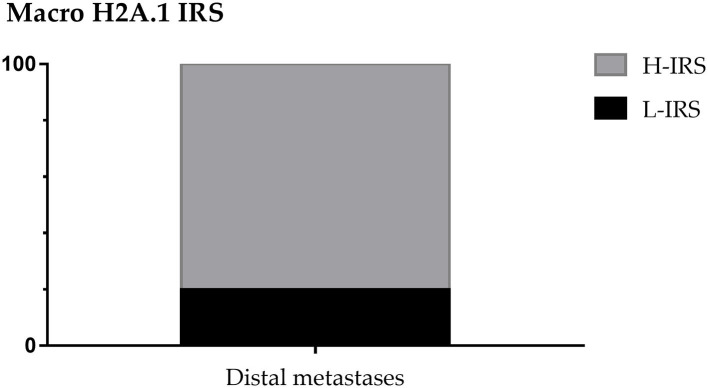
MacroH2A1 Intensity Reactivity Score (IRS) in distal metastases.

**Figure 10:**
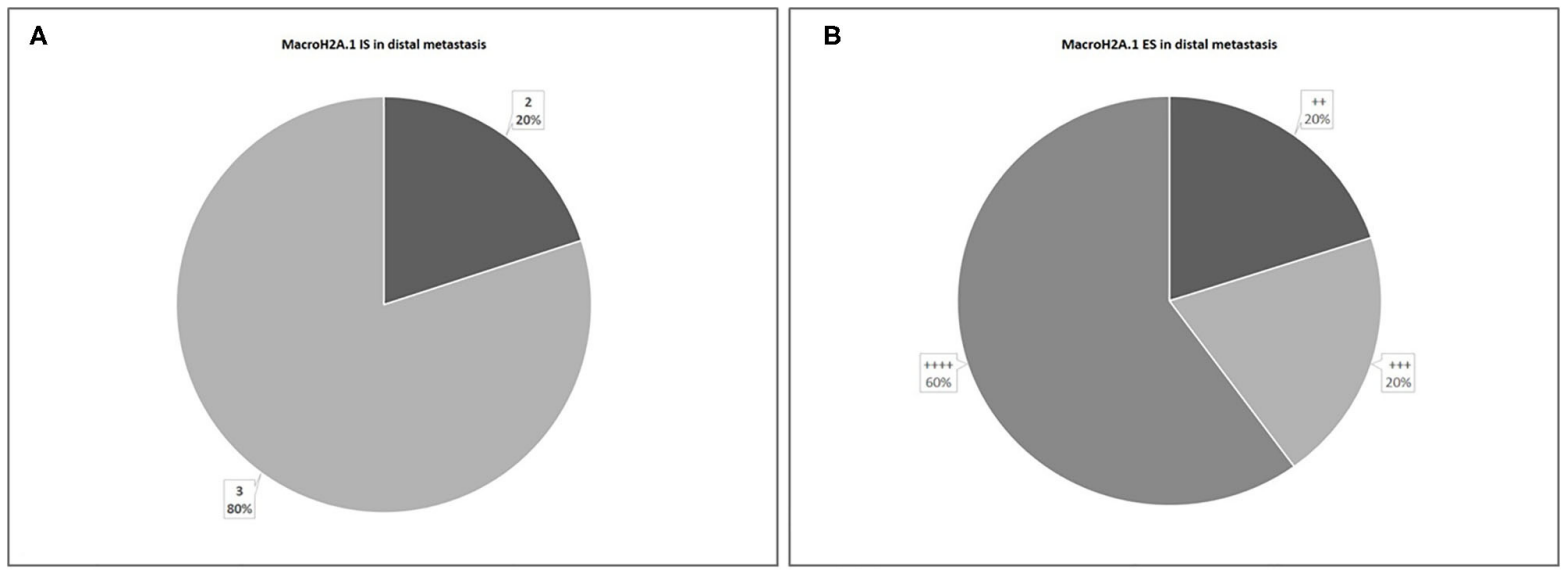
Levels of MacroH2A.1 Intensity of Staining (IS) **(A)** and Extent Score (ES) **(B)** in distal metastases.

About distant metastasis macroH2A.1 H-IRS was observed in 4 cases (80%) and L-IRS in only one case (20%) (Figure 9). MacroH2A1 IS was intermediate in only one case (20%), and intense in 4 cases (80%) (Figure 10A). MacroH2A1 ES was 31–50% in only one case (20%), 51–75% in only one case (20%), and <75% in 3 cases (60%) (Figure 10B). No difference in MacroH2A1 expression was noted between bone and brain metastasis.

## Discussion

Mammary carcinoma is the most frequent malignancy in females and the bone is the most common site of metastases, through mechanisms of disruption of the physiological bone remodeling (32). While similar levels of macroH2A1 isoforms are expressed in normal adult cells, in breast cancer macroH2A1.1 expression tends to reduce (10, 26) and macroH2A1.2 is the predominant form (14). In particular, recent studies have reported the role of macroH2A1.2 in negatively regulating breast cancer-induced osteoclastogenesis (12). MacroH2A1.2 significantly suppresses the production of soluble factors capable of affecting osteoclast differentiation and function in the metastasizing process of breast carcinoma cells to bone (12). Thus, macroH2A1.2 has an essential role in the silencing of the genes that encode osteoclastogenesis and metastasizing related factors in mammary neoplastic cells (12). Initially, macroH2A1.2 is highly expressed and localized in LOX gene, which acts as an upregulator of osteoclast differentiation and bone resorption (12, 33, 34). Once incorporated into LOX gene, macroH2A1.2 utilizes its macrodomain to recruit EZH2 by protein-protein interactions, resulting in the inactivation of LOX gene in neoplastic epithelial cells throughan epigenetic gene silencing process H3K27me3 related (12, 14, 35).

The two other macroH2A isoforms, macroH2A1.1 and macroH2A2, do not influence osteoclastogenesis (12). This is verified because macroH2A1.1's macrodomain can interact with NAD+-derived ligands, such as PAR, while the macrodomains of macroH2A1.2 and macroH2A2 cannot (36). The reduction in macroH2A1.1 is functionally important in cancer and it has effects on the proliferation and metastatic potential of cancer cells (10, 37). In highly proliferative breast cancer has been observed an increased macroH2A1.2/macroH2A1.1 ratio, correlates with poor survival, tumor growth and metastasis (37).

MacroH2A is a differentiation promoting factor that limits the acquisition of malignant characteristics by cancer cells and thus it has generally a tumor suppressor role in cancer (10, 22, 36, 37). Scientific evidences support a tumor suppressive role for macroH2A1.1 and macroH2A2 (10, 22, 23, 35, 37–39), while macroH2A1.2 is an oncogene associated with tumor progression and negative patient outcome (5, 10).

The role of macroH2A1.2 seems to depend on the microenvironment of the specific cancer studied (39). MacroH2A1 either occupies regions marked by H3K27me3 or regions marked by the acetylations. H3K27me3 marks repressed regions of the genome while the panel of the acetylations marks transcriptionally active regions. Thus, two different chromatin environments influence macroH2A1 function (38).

The prognostic role of MacroH2A1 has been investigated in several types of human cancer (7, 10, 12, 21–26, 40) with sometimes conflicting results. The loss of MacroH2A1 isoforms in malignant cutaneous melanoma cells has been correlated with poorer prognosis and gain of an increasing malignant potential through the transcriptional upregulation of CDK8 (25); conversely, in uveal melanoma human tissue samples the immunohistochemical overexpression of MacroH2A1 has been observed both in metastasizing primary tumors and liver metastases, suggesting a role of MacroH2A1 as negative prognostic factor and predictor of the risk of disease progression (21). In addition, a tumor suppressive role of MacroH2A1, particularly of the MacroH2A1.1 isoform, has also been supposed for human prostatic cancer (40).

We investigated immunohistochemical expression of macroH2A1 in primitive breast cancer and metastases. Anti-macroH2A1 antibody used in this study recognizes the known isoforms of macroH2A1 including macroH2A1.2 (longest isoform) and the macroH2A1.1 (shortest isoform). We found that macroH2A over-expression was a predictor of poorer prognosis and higher risk of metastases in breast cancer, and the splice isoform macroH2A1.2 is the most expressed in tumors (5, 10, 14, 37).

Our results showed that in the whole group (*n* = 54) macroH2A1 H-IRS was observed in 29 (54%) primitive breast cancers and L-IRS in 25 (46%).

The 48% of patients showed the presence of metastases, considering the patients with micrometastases as non-metastatic. In 28 non-metastatic primary breast cancers, H-IRS was observed in nine cases (32%) and L-IRS in 19 (68%) while in 26 metastatic primary breast cancers, H-IRS was observed in 20 (77%) breast cancers and L-IRS in 6 (23%). About nodal metastases macroH2A.1 H-IRS was observed in 22 cases (85%) and L-IRS in only four cases (15%). The cases that showed H-IRS in primary breast cancers presented macroH2A1 H-IRS in 21 correlated metastases cases (95%) and macroH2A1 L-IRS in only one metastasis case (5%). The cases that showed L-IRS in primary breast cancers presented macroH2A1 H-IRS in two correlated metastases cases (33%) and macroH2A1 L-IRS in four metastases cases (67%). About distant metastases macroH2A.1 H-IRS was observed in four cases (80%) and L-IRS in only one case (20%).

In conclusion, the immunohistochemical expression of macroH2A1 was more highly expressed in breast cancer with presence of metastases, finding that macroH2A1 is more highly expressed in breast cancer with poorer prognosis. Furthermore, the immunohistochemical expression of macroH2A1 was highly expressed in the metastases in almost all cases; thus, the immunohistochemical expression of macroH2A1 could predict the risk of breast cancer metastasis and thus directing strategies for follow-up and treatment of patients.

## Data Availability Statement

The datasets generated for this study are available on request to the corresponding author.

## Ethics Statement

The studies involving human participants were reviewed and approved by Ethics Committee of Catania 1, Azienda Ospedaliero-Universitaria Policlinico-Vittorio Emanuele. Written, informed consent was obtained from all participants for the publication of any potentially identifiable images or data included in this article.

## Author Contributions

GB and VF contributed to the design and implementation of the study and to the writing of the manuscript. RC, CLor, VR, CLom, EV, CLe, and AI were involved in planning and supervised the study. GB, RC, AI, SV, DR, and GT performed the histopathologic diagnoses and the complete analysis of the surgery material. All authors contributed to the article and approved the submitted version.

## Conflict of Interest

The authors declare that the research was conducted in the absence of any commercial or financial relationships that could be construed as a potential conflict of interest.
